# Flatfish monophyly refereed by the relationship of *Psettodes* in Carangimorphariae

**DOI:** 10.1186/s12864-018-4788-5

**Published:** 2018-05-25

**Authors:** Wei Shi, Shixi Chen, Xiaoyu Kong, Lizhen Si, Li Gong, Yanchun Zhang, Hui Yu

**Affiliations:** 1 College of Life Science, Foshan University, Foshan, 528231 Guangdong China; 2 CAS Key Laboratory of Tropical Marine Bio-resources and Ecology, South China Sea Institute of Oceanology, Chinese Academy of Sciences, 164 West Xingang Road, Guangzhou, 510301 China; 3 University of Chinese Academy of Sciences, Beijing, 100049 China

**Keywords:** Mitochondrial genomes, Psettodes, Pleuronectiformes, Molecular clock, Phylogeny

## Abstract

**Background:**

The monophyly of flatfishes has not been supported in many molecular phylogenetic studies. The monophyly of Pleuronectoidei, which comprises all but one family of flatfishes, is broadly supported. However, the Psettodoidei, comprising the single family Psettodidae, is often found to be most closely related to other carangimorphs based on substantial sequencing efforts and diversely analytical methods. In this study, we examined why this particular result is often obtained.

**Results:**

The mitogenomes of five flatfishes were determined. Select mitogenomes of representative carangimorph species were further employed for phylogenetic and molecular clock analyses. Our phylogenetic results do not fully support *Psettodes* as a sister group to pleuronectoids or other carangimorphs. And results also supported the evidence of long-branch attraction between *Psettodes* and the adjacent clades*.* Two chronograms, derived from Bayesian relaxed-clock methods, suggest that over a short period in the early Paleocene, a series of important evolutionary events occurred in carangimorphs.

**Conclusion:**

Based on insights provided by the molecular clock, we propose the following evolutionary explanation for the difficulty in determining the phylogenetic position of *Psettodes*: The initial diversification of *Psettodes* was very close in time to the initial diversification of carangimorphs, and the primary diversification time of pleuronectoids, the other suborder of flatfishes, occurred later than that of some percomorph taxa. Additionally, the clade of *Psettodes* is long and naked branch, which supports the uncertainty of its phylogenetic placement. Finally, we confirmed the monophyly of flatfishes, which was accepted by most ichthyologists.

**Electronic supplementary material:**

The online version of this article (10.1186/s12864-018-4788-5) contains supplementary material, which is available to authorized users.

## Background

Flatfishes (order Pleuronectiformes) are divided into two suborders: Psettodoidei, with one family, one genus (*Psettodes*); and Pleuronectoidei (hereafter pleuronectoids), with 13 families, approximately 128 genera. The eyes of these adult fishes are uniquely located on one side of the body. The absence of transitional species of flatfishes offered an early challenge to theories of evolutionary change through the accumulation of a series of small steps [1].

The study of flatfish fossils has been ongoing. Schwarzhans [2] documented the recent and fossil otoliths of the order. Chanet [3] summarized studies regarding the fossils of this order. Friedman [4] named †Heteronectes and re-studied †Amphistium. Friedman [5] placed the Eocene crown-group flatfish †Joleaudichthys in Psettodoidei and two other Eocene crown-group fossils, †Numidopleura and †Eobothus, within Pleuronectoidei. [1].

Two distinct views regarding the origin of flatfishes have been presented [6]. One, proposed by Kyle [7] and Chabanaud [8], considers that all major flatfish lineages were independent offshoots of an evolving “pre-perciform” lineage and treats *Psettodes* as the descendant of a recent percoid ancestor. However, Chapleau [6] and a number of ichthyologists believe that flatfishes have a “lower-percoid” origin. *Psettodes* has been suggested to be the most “primitive” flatfish [9–11]. The “lower-percoid” origin of flatfishes was recently confirmed by molecular data and is now widely accepted by teleost ichthyologists [12–17].

Moreover, recent studies have confirmed that the most closely related organisms to flatfishes are percomorph taxa, predominantly carangiforms and istiophoriforms, including latids, carangoids, billfishes, moonfish, swordfish, barracudas, archerfishes, snook and threadfins (this group is referred to as the cara group hereafter) [12–15, 18–24]. These taxa and flatfishes were initially collectively referred to as clade L by Chen et al. [17]. Li et al. [23] named this clade Carangimorpha, which was soon after referred to as Carangimorphariae (hereafter, carangimorphs) by Betancur-R et al. [19, 20], and conferred upon it a new taxonomic rank in a revised classification of bony fishes.

While the monophyly vs. polyphyly of the pleuronectiforms remains disputed, much molecular research on teleostean phylogenies has involved the flatfishes [12, 14, 15, 17, 21–23, 25]. Most studies focus on the phylogenetic status of flatfishes among teleosteans (especially percomorphs), but only representatives of pleuronectiforms have been included [13, 22]. Recently, molecular studies with broader taxonomic representation for this prominent group have been performed [12, 18, 19, 26], but the results are highly divergent, especially those presented by two major research groups, Betancur-R. et al. [19, 27] and Campbell et al. [18, 28], who strongly debate flatfish monophyly. Campbell et al. [26] used whole mitochondrial genome sequences to examine the phylogenetic affinities of the flatfishes (Pleuronectiformes) and obtained only weak support for the monophyly of Pleuronectiformes.

In the last ten years, several large-scale fish phylogenetics projects broadly representing the carangimorphs have been performed, which strongly support the monophyly of Pleuronectoidei [18–20, 27]. However, whether the other suborder of flatfishes, Psettodoidei, with close relationship to Pleuronectoidei remains unclear [12, 20, 23, 24, 29]. Betancur-R. et al. [19] conducted a thorough investigation of the phylogeny of flatfishes and their position among percomorphs by combining high genetic coverage (20 loci; ca. 20 kbp) with dense taxonomic breadth (214 taxa), including all putative flatfishes and a diverse percomorph outgroup. The majority of concatenation topologies provide evidence that flatfish has a single evolutionary origin, although a minority of analyses have inferred a non-monophyletic Pleuronectiformes, with varying placement of *Psettodes* and pleuronectoid clades among carangimorphs. As mentioned above, these authors support the monophyly of flatfishes. However, Campbell et al. [18] rapidly cast doubt on the monophyly of flatfishes based on six nuclear genes and extensive taxonomic sampling, including flatfishes and potential close relatives (approximately 90 taxa). Their results were most consistent with a non-monophyletic Pleuronectiformes, with *Psettodes* consistently excluded from other flatfishes and placed among other carangimorphs. Soon thereafter, Campbell et al. [28] and Betancur-R. et al. [27] launched a continuous debate based on more comprehensive data or complete (mitochondrial genome) mitogenome data. This issue has become a hot topic, and there is no consensus. Harrington et al. [30] presented a high-resolution phylogeny using a sequence dataset comprising more than 1000 ultraconserved DNA element loci covering 45 carangimorphs that unequivocally supports flatfish monophyly and a single origin of asymmetry. It remains unclear why so many phylogenetic analyses based on different datasets still have failed to clarify the issue of flatfish monophyly.

Currently, the key problem challenging the monophyly of flatfishes is the phylogenetic placement of *Psettodes.* What factors are responsible for the inconsistent phylogenetic position of the *Psettodes* clade? In this study, the mitogenomes of five flatfishes, *Psettodes erumei, Samaris cristatus, Achirus lineatus, Trinectes maculatus* and *Cynoglossus nanhaiensis*, were determined, and species data from all 13 flatfish families were compiled. Select mitogenomes of representative carangimorph species were employed for phylogenetic and molecular clock analyses. Based on our evaluation of the evolutionary history of carangimorphs, particularly the evolutionary events during the period of emergence of the *Psettodes* ancestor, we explain why different molecular phylogenetic studies are so divided on the issue of flatfish monophyly.

## Methods

### Sampling, DNA extraction, amplification and sequencing

The *Psettodes erumei, Samaris cristatus, Achirus lineatus, Trinectes maculatus* and *Cynoglossus nanhaiensis* specimens used in this study were collected from a seafood market. A summary of the primer sequences and optimized PCR conditions used for amplifying the metagenomes are presented in Additional file 1: Table S1. The obtained PCR products were purified and sequenced in both directions with an ABI 3730 DNA sequencer (Applied Biosystems, USA).

The sequenced fragments were assembled into the mitochondrial genome using CodonCode Aligner v3.5.4 (CodonCode Corporation) and BioEdit v7.2.5 [31]. Annotation and boundary determination for protein-coding genes and ribosomal RNA genes were performed using NCBI-BLAST (http://blast.ncbi.nlm.nih.gov) and tRNAscan-SE [32], with the cut-off values set to 1 when necessary.

Dense taxonomic sampling can reduce the effects of systematic biases, such as long-branch attraction, on phylogenetic inference [33]. In this study, the complete mitochondrial genome sequences from 50 fishes were employed for phylogenetic analysis. Five new complete mitogenomes were obtained in the present study, and the sequences of the remaining 45 species were retrieved from GenBank. The 47 carangimorphs that were included represented all 13 available families of flatfishes (30 species) as well as all 11 previously studied families (17 species) of percomorphs from Carangimorphariae. *Beryx splendens* (Beryciformes)*, Myripristis berndti* (Holocentriformes) and *Channa maculata* (Anabantiformes) were used as outgroup (Table 1).

**Table 1:** Description of the 50 species included in this study. Classification follows Nelson [1]

Taxa	Order	Family	Species	Accession NO
Flatfishes	Pleuronectiformes	Psettodidae	*Psettodes erumei*	NC_020032
		Citharidae	*Citharoides macrolepis*	/
			*Citharoides macrolepidotus*	NC_024948
			*Lepidoblepharon ophthalmolepis*	NC_024952
		Scophthalmidae	*Psetta maxima*	NC_013183
		Paralichthyidae	*Pseudorhombus cinnamoneus*	NC_022447
			*Pseudorhombus dupliciocellaius*	NC_029323
			*Cyclopsetta fimbriata*	NC_024950
		Pleuronectidae	*Platichthys stellatus*	NC_010966
			*Pleuronichthys cornutus*	NC_022445
			*Hippoglossus stenolepis*	NC_009710
		Bothidae	*Crossorhombus azureus*	NC_022446
			*Lophonectes gallus*	NC_030367
			*Bothus myriaster*	NC_030365
		Poecilopsettidae	*Poecilopsetta natalensis*	/
		Rhombosoleidae	*Pelotretis flavilatus*	NC_026284
			*Colistium nudipinnis*	NC_023447
			*Peltorhamphus novaezeelandiae*	NC_023448
		Achiropsettidae	*Neoachiropsetta milfordi*	NC_024953
		Samaridae	*Samaris cristatus*	NC_025903
			*Samaris cuslatus*	NC_024263
		Achiridae	*Achirus lineatus*	NC_023768
			*Trinectes maculatus*	NC_023769
		Soleidae	*Zebrias quagga*	NC_023225
			*Solea ovata*	NC_024610
			*Heteromycteris japonicus*	NC_024921
		Cynoglossidae	*Paraplagusia blochii*	NC_023228
			*Symphurus plagiusa*	JQ639061
			*Symphurus orientalis*	NC_027656
			*Cynoglossus nanhaiensis*	MH317761
Cara-group	Holocentriformes	Holocentridae	*Myripristis berndti*	AP002940
	Carangiformes	Coryphaenidae	*Coryphaena equiselis*	AB355907
			*Coryphaena hippurus*	AB355908
		Rachycentridae	*Rachycentron canadum*	NC_011219
		Echeneidae	*Echeneis naucrates*	NC_022508
		Carangidae	*Carangoides armatus*	NC_004405
			*Seriola dumerili*	NC_016870
		Menidae	*Mene maculata*	AB355909
	Istiophoriformes	Sphyraenidae	*Sphyraena barracuda*	NC_022484
			*Sphyraena japonica*	NC_022489
		Xiphiidae	*Xiphias gladius*	NC_012677
		Istiophoridae	*Makaira indica*	NC_012675
			*Istiophorus albicans*	NC_022478
			*Istiophorus platypterus*	NC_012676
	Anabantiformes	Channidae	*Channa maculata*	JX978724
	Perciformes	Polynemidae	*Polydactylus plebeius*	NC_026235
			*Polydactylus sextarius*	NC_027088
Outgroup	Beryciformes	Berycidae	*Beryx splendens*	AP002939
	Perciformes	Latidae	*Lates calcarifer*	NC_007439
		Toxotidae	*Toxotes chatareus*	NC_013151

### Phylogenetic analysis

In all 50 complete mitochondrial genomes, the first (1_N_), second (2_N_) and third codon positions of twelve coding sequences (*ND6* excluded due to compositional heterogeneity), 2 rRNAs (R) and 22 tRNAs (T) were concatenated separately and aligned with Clustal X2 [34], and ambiguous sequences were eliminated using Gblock [35]. To determine whether saturation existed in the alignments, the substitution saturation and the substitution vs. Tamura-Nei (TN93) genetic distance in pairwise comparisons were tested with DAMBE [36]. The number of transitional (TS) and transversional (TV) differences in pairwise comparisons increased with increasing evolutionary distance in all aligned datasets. Third codon positions showing saturation were also observed. Thus, the third codon position sequences were defined only as purines and pyrimidines (3_RY_) [26].

Partitioned 1_N_2_N,_ 1_N_2_N_RT, 1_N_2_N_3_RY_ and 1_N_2_N_3_RY_RT sequences were employed to perform Bayesian inference (BI) analyses in MrBayes 3.2 [37] based on a partitioning strategy for complete mitogenome data described by previous phylogenetic studies with complete mitogenomes [16, 26, 38]. The best-fit models of nucleotide substitution for each of the sequences were selected under different partitioning strategies using MrModelTest 2.1 [39]. Bayesian phylogenetic analyses were performed using “Lset” and “Prset”, and the program was allowed to converge on the best estimates of the model parameters. Other parameter settings were as follows: Each Markov chain was initiated from a random tree and run for 5.0 × 10^6^ generations, with every 100th generation being sampled from the chain to assure independence of the samples. Four chains (three heated (temperature = 0.5) and one cold) were run simultaneously using the Metropolis-coupled Markov chain Monte Carlo (MCMCMC) method to enhance the mixing capabilities of the Markov chains. To examine whether stationarity had been reached, the fluctuating values of likelihood and all the phylogenetic parameters were monitored graphically, and simulation analysis was performed twice, starting from different random trees, until the average standard deviation of split frequencies fell below 0.01.

Partitioned 1_N_2_N,_ 1_N_2_N_RT, 1_N_2_N_3_RY_ and 1_N_2_N_3_RY_RT information was also input into RAxML software [40] for maximum likelihood (ML) analysis. A general time-reversible model with sites following a discrete gamma distribution (GTRGAMMA) was used. A rapid bootstrap (BS) analysis was conducted with 200 replications, and the software produced a best-scoring ML tree with BS probabilities.

### Molecular clocks

The mtDNA datasets were partitioned as follows: concatenated codon positions first (1_N_), second (2_N_), 2 rRNA (R) and 22 tRNA (T). We employed the 1_N_2_N_RT data-coding scheme in the divergence time analysis. BI under various relaxed-clock models, implemented with MultiDivTime [41] and BEAST v1.7.5 [42], was used to perform molecular dating.

Under the MultiDivTime approach, branch lengths were estimated using ESTBRANCHES, with a fixed tree topology in which the flatfishes were constrained to cluster into a single clade. Next, MULTIDIVTIME was employed to estimate the prior and posterior ages of branching events, standard deviations and 95% credibility intervals. The Markov chain was run for 10,000,000 generations and sampled every 100 generations after an initial burn-in period of 1000,000 cycles. Other parameters were as follows: The priors for the mean and standard deviation of the ingroup root age, rttm and rttmsd, were set to equivalents of 70 million years and 10 million years (i.e., rttm = 0.7, rttmsd = 0.1), respectively. The prior mean and standard deviation for the Gamma distribution describing the rate at the root node (rtrate and rtratesd) were both set to 0.34. These values were based on the median of the substitution path lengths between the ingroup root and each terminal and divided by rttm (as suggested by the author). The prior mean and standard deviation for the Gamma distribution of the parameter controlling rate variation over time (i.e., brownmean and brownsd) were both set to 2.85.

In the BEAST analyses, the uncorrelated lognormal model was used to describe the relaxed clock, while GTR + I + G was used to describe the substitution model for the four partitions of the dataset. The Yule process was employed to describe speciation. The constrained tree in which all flatfishes were clustered together was used as the input topology. The means and standard deviations of the lognormal distribution for each calibration point were chosen so that 95% of the probability lay within the minimum and the maximum boundaries, and the means were the arithmetical medians of the intervals. An MCMC test run with 10^6^ generations was first performed to optimize the scaling factors of the priori function. For every individual analysis, the final MCMC chain was run twice for 30 million generations, sampled every 1000 generations. The burn-in and convergence of the chains were determined with Tracer 1.3 [42].

Four calibration points were used in MultiDivTime and BEAST analyses, as performed by Near et al. [29]: (1) date of the most recent common ancestor (MRCA) of Carangidae, Rachycentridae and Echeneidae: 55.8 million years ago (Mya) as the minimal age offset and 63.9 Mya as the 95% soft upper boundary (BEAST: mean = 0.776, SD = 0.8 and offset = 55.8; MultiDivTime: 0.55–0.65); (2) date of the MRCA of Rachycentridae and Echeneidae: 30.1 Mya as the minimal age offset and 34.5 Mya as the 95% soft upper boundary (BEAST: mean = 0.165, SD = 0.8 and offset = 30.1; MultiDivTime: 0.3–0.35); (3) date of the MRCA of Soleidae and Cynoglossidae: 40.4 Mya as the minimal age offset and 50 Mya as the 95% soft upper boundary (BEAST: mean = 0.946, SD = 0.8 and offset = 40.4; MultiDivTime: 0.4–0.5); and (4) date of the MRCA of Paralichthyidae, Bothidae and Pleuronectidae: 30.0 Mya as the minimal age offset and 34.4 Mya as the 95% soft upper boundary (BEAST: mean = 0.165, SD = 0.8 and offset = 30; MultiDivTime: 0.3–0.35).

## Results

### Phylogenetic analysis based on mitochondrial genomes

**Fig. 1:**
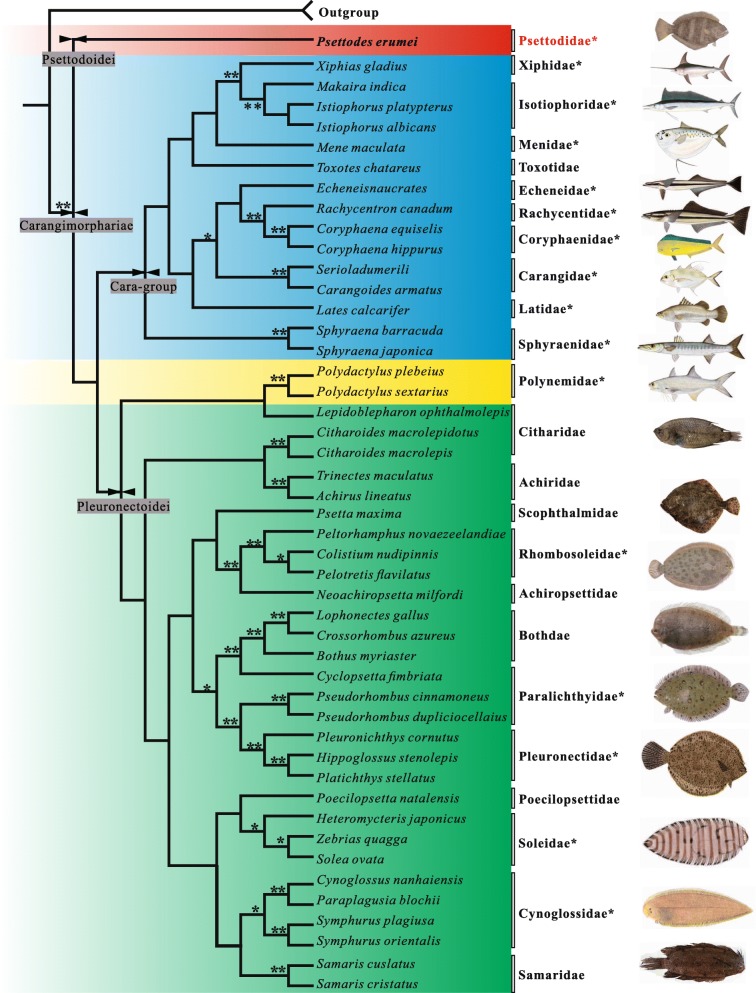
Maximum likelihood (ML) tree generated in RAxML version 8.0.0 under a GTR + Γ model of nucleotide evolution. The sequences of the first and second codon positions of coding sequences, the third codon position mitochondrial sequences (as purines and pyrimidines), and rRNA and tRNA (1_N_2_N_3_RY_RT) were used; the *Psettodes* background is red, while the cara-group taxon background is blue and yellow, and the Pleuronectoidei background is green. * on the branches indicates > 50% BPs. ** indicates 100% BPs. The fish freehand images marked with asterisk were provided by Bernard P.H. YAU, and the other photographs were taken by Kong XY

Eight phylogenetic topologies were obtained: 1_N_2_N-_BI_,_ 1_N_2_N_RT-BI, 1_N_2_N_3_RY-_BI_,_ 1_N_2_N_3_RY_RT-BI, 1_N_2_N-_ML_,_ 1_N_2_N_RT-ML, 1_N_2_N_3_RY-_ML (Additional file 2: Figure S1 a-g) and 1_N_2_N_3_RY_RT-ML (Fig. 1). The eight topologies showed most flatfish families were monophyletic, including Pleuronectidae, Bothidae, Rhombosoleidae, Samaridae, Achiridae, Soleidae, and Cynoglossidae. The Citharidae and Achiriae clades were sisters to the other pleuronectoids as a whole, while three distinct clades were formed in the pleuronectoids: one stable clade included Paralichthyidae, Pleuronectidae and Bothidae, another included Poecilopsettidae, Samaridae, Soleidae and Cynoglossidae, and the third included Rhombosoleidae and Achiropsettidae (Fig. 1). However, not all flatfish families always clustered together; some cara-group taxa were inserted in the pleuronectoid clade and especially the Citharidae clade (Fig. 1 and Additional file 2: Figure S1 a-g). The close relationship between these cara-group taxa and Citharidae was not supported by high PPs or BPs in the topologies, which is consistent with the results of previous studies [18, 19, 26]. Without inclusion of these cara-group taxa, all the topologies supported the monophyly of pleuronectoids (Additional file 2: Figure S1a-g).

Most significantly, the placement of the *Psettodes* clade differed greatly among the eight topologies. The sister relationships of *Psettodes* in each topology are listed in Table 2. The clustering of the *Psettodes* clade with pleuronectoids arose only in the 1_N_2_N_-BI topology (Additional file 2: Figure S1a), while in four other topologies, the *Psettodes* clade was clustered with different cara-group families or the main clade containing all cara-group families (Additional file 2: Figure S1c-e, g). In the other three topologies, among which one tree was based on the most informative datasets (Fig. 1), the *Psettodes* clade was the first to diverge from the entire carangimorph clade (Fig. 1; Additional file 2: Figure S1 b, f).

**Table 2:** Summary of phylogenetic sister relationships of *Psettodes* in the eight analyses

Method	Partition	*Psettodes* sister relationship to	Phylogenetic tree
Bayes	1_N_2_N_	Pleuronectiformes^a^	Additional file 2: Figure S1a
	1_N_2_N_3_RY_	Menidae+Latidae+Toxotidae+Istiophoridae+Xiphiidae+Carangidae	Additional file 2: Figure S1b
	1_N_2_N_ RT	Cara-group+Pleuronectiformes	Additional file 2: Figure S1c
	1_N_2_N_3_RY_ RT	Cara group	Additional file 2, Figure S1d
RAxML	1_N_2_N_	Latidae+Toxotidae+Istiophoridae+Xiphiidae	Additional file 2: Figure S1e
	1_N_2_N_3_RY_	Menidae+Toxotidae+Istiophoridae+Xiphiidae	Additional file 2: Figure S1 g
	1_N_2_N_ RT	Cara-group+Pleuronectiformes	Additional file 2: Figure S1f
	1_N_2_N_3_RY_ RT	Cara-group+Pleuronectiformes	Figure 1

^a^Polynemidae and Sphyraenidae are excluded.

### Origin and evolution of *Psettodes*

**Fig. 2:**
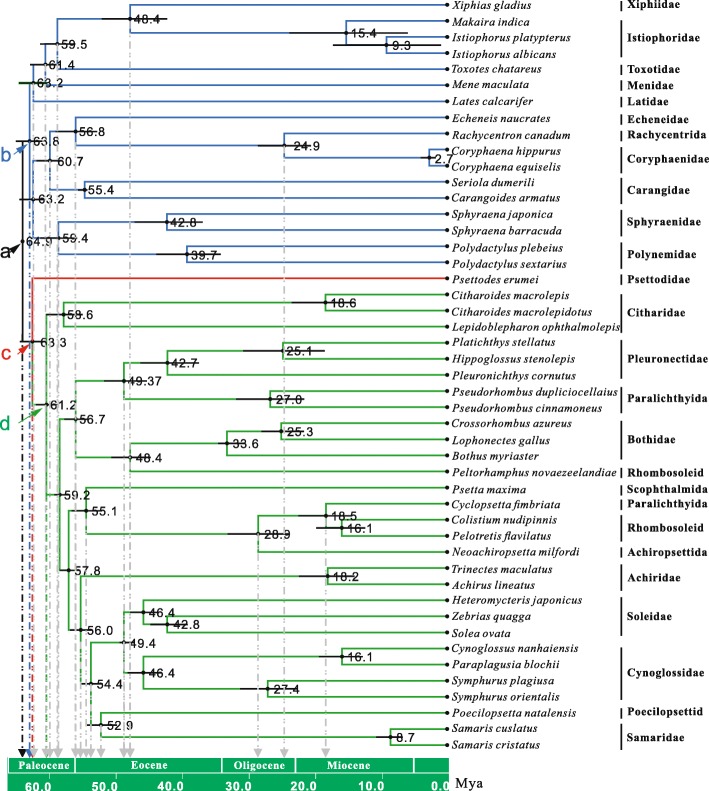
Divergence times determined using a partitioned Bayesian relaxed molecular clock method. Only partial sequences of the first and second codon positions of coding sequences, rRNA, and tRNA (1_N_2_N_RT) were used. The numbers beside the nodes indicate the divergence time estimated by MultiDivTime in millions of years (Mya). The horizontal bar at the node indicates the 95% highest probability density; a: the first dichotomic time of carangimorphs, b: the first dichotomic time of cara-group taxa, c: the first dichotomic time of flatfish taxa and d: the first dichotomic time of pleuronectoid taxa. Gray downward lines indicate the dichotomic time at the level of family

We used our mitogenome dataset to estimate divergence times for major lineages of carangimorphs employing methods implemented in MultiDivTime [41] and BEAST [42]. Overall, the two dating methods yielded similar results, although some divergence time estimates were slightly different between the two methods. In the MultiDivTime analysis, the partitioned Bayesian approach estimated that the dichotomic time between the flatfishes and cara-group taxa was in the early Paleocene, approximately 64.9 Mya (Fig. 2, node a), and suggested that modern *Psettodes* originated during the same period in the early Paleocene, approximately 63.3 Ma (Fig. 2, node c). The dichotomic time for pleuronectoids was in the middle Paleocene, approximately 61.2 Mya (Fig. 2, node d), followed by subsequent expansion to form the 12 extant families. In the overall evolutionary process, *Psettodes* was not observed to experience any species expansion, with a single genus being maintained for approximately 60 million years.

Accordingly, the initial diversification of the cara group also occurred in the early Paleocene, approximately 63.8 Mya (Fig. 2, node b). After approximately a half Mya, two clades of cara-group taxa underwent further diversification, which indicated that the cara-group taxa experienced speciation expansion in the early Paleocene.

We also mapped all the diversification times at the family level. Most divergences were concentrated within the Paleocene and early Eocene, and for a long time thereafter, there were only a couple of diversification events at the family level (Fig. 2, gray lines). The mapping reveals that during the Paleocene and early Eocene, both flatfishes and the cara-group taxa experienced diversification.

## Discussion

### Unstable phylogenetic placement of *Psettodes*

To balance the phylogenetic tree and reduce branch attraction error, we supplemented the complete mitogenome sequence data for Achiridae (*Achirus lineatus, Trinectes maculatus*) and Samaridae (*Samaris cristatus, Samaris cuslatus*) and ensured that species data were available from all 13 flatfish families. An analytical strategy of balanced taxon sampling was chosen, and only 47 species from Carangimorphariae were intensively reselected, including 17 cara-group fishes and 30 flatfishes, with only 1–4 species being sampled from the carangimorph families in this phylogenetic analysis (Table 1). Based on the partitioning strategy for complete mitogenome data used by Campbell [26], four representative partitioned datasets (1_N_2_N,_ 1_N_2_N_RT, 1_N_2_N_3_RY_, and 1_N_2_N_3_RY_RT) were selected and subjected to RAxML (ML) analyses and BI.

The phylogenetic topologies constructed this study are slightly different from those of previous studies. For example, Betancur-R. et al. [19] concluded that flatfishes are a monophyletic group based on the results of a majority of their concatenation topologies. In contrast, Campbell et al. [18] found that *Psettodes* consistently clustered within the cara-group taxa and confirmed the paraphyly of Pleuronectiformes. In our study, most of the topologies showed that *Psettodes* did not group with the other flatfishes, but these topologies did not consistently group *Psettodes* with fixed cara-group fishes. An additional finding of the present study that has not been reported by previous work was that three of our topologies excluded *Psettodes* not only from other flatfishes but also from cara-group fish, representing the first split from all carangimorphs (Fig. 1 and Table 2). Thus, the topologies do not consistently support *Psettodes* as the sister to pleuronectoids, the sister to cara*-*group taxa or the sister to all other carangimorphs.

### Reason for the inconsistent phylogenetic placement of *Psettodes*

If important evolutionary events occurred in the period of *Psettodes* divergence, they would have influenced branch attraction in the phylogenetic analysis. Molecular clock analysis can not only provide the origin timing of the *Psettodes* lineage based on sequence information from modern species but also indicate the evolutionary events that occurred in closely related lineages in the same period. Important evolutionary events can be the cause of branch attraction in phylogenetic analyses. The chronograms derived from the Bayesian relaxed-clock methods provide following insights. Within a short period in the early Paleocene, a series of important evolutionary events occurred in Carangimorphariae, including the initial divergence between pleuronectiforms and cara-group taxa (Fig. 2, node a); the origin of *Psettodes* (Fig. 2, node c); and the subsequent speciation expansion of the cara group (Fig. 2, node b). Restoring the evolutionary course, *Psettodes* and some taxa of the cara group originated, and speciation expansion occurred in the cara group shortly thereafter during a short period in the initial diversification of carangimorphs. At meantime, none of the flatfishes underwent speciation expansion. Only in a later period of the middle Paleocene pleuronectoids did undergo speciation expansion (Fig. 2, node d).

From the initial origin of *Psettodes* to the modern era, a duration of over approximately 60 million years, no diversification has occurred within the lineage of *Psettodes*, with the *Psettodes* clade representing as a long, naked branch in phylogenies. The only genus *Psettodes* in family Psettodidae comprises *Psettodes belcheri* Bennett 1831, *Psettodes bennettii* Steindachner 1870 and *Psettodes erumei* (Bloch & Schneider 1801). We constructed a phylogenetic tree based on 16S rRNA sequences (Additional file 3: Figure S2), and the topology showed all three *Psettodes* species as being very closely related and a long and naked branch leading to the *Psettodes* clade. The long and naked branch is likely the result of long-branch attraction (LBA), which can lead to anomalous groupings of long lineages in a phylogeny. Siddall and Whiting (1999) indicated that if the phylogenetic relationships among taxa change when LBA taxa are systematically removed from the analysis one at a time, this is good evidence of LBA. Thus, we removed the LBA *Psettodes* taxa and re-constructed the phylogenetic tree (Additional file 4: Figure S3). The phylogenetic relationships changed markedly: *Polydactylus plebeius* and *Polydactylus sextarius*, which formerly clustered with flatfishes in Fig. 1, now clustered with the cara-group taxa. In addition, the cara-group clade showed a strong ability to attract the *Psettodes* clade because the time of speciation expansion for the cara group was very early and very close to the time of origin of the *Psettodes* lineage (Fig. 2, node b & c), making the cara-group clade both “weighty” and closely related to the *Psettodes* clade. In this scenario, the pleuronectoid clade exhibits no advantage over the cara-group clade in the competition for attraction of the *Psettodes* clade in the phylogenetic analysis.

The above conclusions are supported by the timetree based on a Bayesian relaxed clock constructed by Campbell et al. [18] in which flatfishes were not monophyletic, and *Psettodes* was sister to some cara-group taxa; however, the origin of the *Psettodes* clade and the divergence between the cara-group lineage and the pleuronectoid lineage were very close in time, as evidenced by the short branch lengths, after the first divergence of the carangimorphs.

## Conclusions

The monophyly of Pleuronectoidei is strongly supported by many large-scale fish phylogenetics studies, but whether the other suborder of flatfishes, Psettodoidei, is closely related to Pleuronectoidei has been difficult to determine. In this study, we examined why flatfish monophyly is difficult to ascertain.

In conclusion, the inconsistent phylogenetic results regarding the placement of *Psettodes* could be due to the origin of the *Psettodes* lineage occurring very close in time to the initial diversification of Carangimorphariae or to the initial diversification of pleuronectoids (the other suborder of flatfishes) occurring later than that of the cara group. In these scenarios, the cara-group clades would inevitably affect the clustering of *Psettodes* in phylogenetic analyses. The other important reason for the uncertain phylogenetic placement of the *Psettodes* clade has a long and naked branch, which is likely an artifact of LBA. With the ongoing advances in phylogenomics and high-throughput sequencing at the genome level, phylogenies will become more accurate, and the question of flatfish monophyly will eventually be answered.

## Additional files

Additional file 1: **Table S1.** Primers used to amplify fragments of the *Psettodes erumei, Samaris cristatus, Achirus lineatus, Trinectes maculatus* and *Cynoglossus nanhaiensis* mitochondrial genomes (DOCX 18 kb)

Additional file 2: **Figure S1.** **a** Relationships of Carangimorphariae taxa inferred from the Bayesian analysis v3.2 of the 50 taxa of dataset 1_N_2_N_. Numbers above or below the branches indicate Bayesian posterior probabilities (shown as percentages). **b** Relationships of Carangimorphariae taxa inferred from the Bayesian analysis version 3.2 of 50 taxa of dataset 1_N_2_N_3_RY_. Numbers above or below internal branches indicate Bayesian posterior probabilities (shown as percentages). **c** Relationships of Carangimorphariae taxa inferred from the Bayesian analysis version 3.2 of the 50 taxa of dataset 1_N_2_N_RT. Numbers above or below internal branches indicate Bayesian posterior probabilities (shown as percentages). **d** Relationships of Carangimorphariae taxa inferred from the Bayesian analysis version 3.2 of 50 taxa of dataset 1_N_2_N_3_RY_RT. Numbers above or below internal branches indicate Bayesian posterior probabilities (shown as percentages). **e** A maximum likelihood (ML) tree generated in RAxML version 8.0.0 under a GTR + Γ model of nucleotide evolution. The 50 taxa mitogenomes were partitioned by codon position (1_N_2_N_). **f** A maximum likelihood (ML) tree generated in RAxML version 8.0.0 under a GTR + Γ model of nucleotide evolution. The 50 taxa mitogenomes were partitioned by codon position with the third codons recoded (1_N_2_N_3_RY_). **g** A maximum likelihood (ML) tree generated in RAxML version 8.0.0 under a GTR + Γ model of nucleotide evolution. The 50 taxa mitogenomes were partitioned by codon position, rRNA, and tRNA (1_N_2_N_RT). (DOCX 2386 kb)

Additional file 3: **Figure S2.** Maximum likelihood (ML) tree generated in RAxML version 8.0.0 under a GTR + Γ model of nucleotide evolution. The sequences of the 16S gene were used; the cara-group background is blue, the Pleuronectoidei background is green, and the *Psettodes* background is red. (EPS 7422 kb)

Additional file 4: **Figure S3.** Maximum likelihood (ML) tree generated in RAxML version 8.0.0 under a GTR + Γ model of nucleotide evolution without *Psettodes* taxa. The sequences of the first and second codon positions of coding sequences, the third codon position mitochondrial sequences (as purines and pyrimidines), rRNA and tRNA (1_N_2_N_3_RY_RT) were used. (EPS 7323 kb)

## Abbreviations

1_N_2_N_: Concatenated dataset of the first, second codon positions of twelve coding sequences (*ND6* excluded due to compositional heterogeneity)
1_N_2_N_3_RY_: Concatenated dataset of the first, second and third (defined only as purines and pyrimidines codon) of twelve coding sequences (*ND6* excluded due to compositional heterogeneity)
1_N_2_N_3_RY_RT: Concatenated dataset of the first, second and third positions (defined only as purines and pyrimidines codon) of twelve coding sequences (*ND6* excluded due to compositional heterogeneity), 2 rRNAs and 22 tRNAs
1_N_2_N_RT: Concatenated dataset of the first, second codon positions of twelve coding sequences (*ND6* excluded due to compositional heterogeneity), 2 rRNAs and 22 tRNAs
BI: Bayesian inference
BP: Bootstrap support
LBA: Long-branch attraction
MCMC: Markov chain Monte Carlo
ML: Maximum likelihood
MRCA: Most recent common ancestor
mtDNA: Mitochondrial deoxyribonucleic acid
Mya: Million years ago
PCR: Polymerase chain reaction
PP: Posterior probability
rRNA: Ribosomal Ribonucleic Acid
tRNA: Transfer Ribonucleic Acid
