# Supplementary material for: Flatfish monophyly refereed by the relationship of Psettodes in Carangimorphariae
Source: BMC Genomics. 2018 May 25;19:400. doi: 10.1186/s12864-018-4788-5 (PMC5970519; doi:10.1186/s12864-018-4788-5)
Supplement: Supplementary file 1 — Table S1. Primers used to amplify fragments of the Psettodes erumei, Samaris cristatus, Achirus lineatus, Trinectes maculatus and Cynoglossus nanhaiensis mitochondrial genomes (DOCX 18 kb) [file 12864_2018_4788_MOESM1_ESM.docx]

Table.S1 Primers used to amplify fragment of the *Psettodes erumei, Samaris cristatus, Achirus lineatus, Trinectes maculatus* and *Cynoglossus nanhaiensis* Mitochondrial Genomes

|  | Forward primer | Sequences (5′-3′) | Reverse primer | Sequences (5′-3′) |
| --- | --- | --- | --- | --- |
| *Psettodes erumei* | PN-Z-15-1 | CCAAGGGAAATCAGCAGT | PN-F-2671-1 | CAAGGGAGGGTGAGGTTT |
|  | PN-Z-15-2 | AACGGCGTAAAGAGTGGT | PN-F-2671-2 | TAAACAAGGGAGGGTGAG |
|  | PN-Z-Thr-1 | TGGCAGGCTGATTAGAAA | PN-F-12S-1 | TCATAAGGGAATCGTGGG |
|  | PN-Z-Thr-2 | CTCATACCTCTGGCTCCC | PN-F-12S-2 | TGTGGGCTAAGTGGGTTG |
|  | PN-COI-F | AGGTTGGCTAAGTTCGGC | PN-COI-R | ACACCGCCTTCTTTGACC |
|  | PN34-47-F | AGCACCTAAGCAGAGTCC | PN34-47-R | TTTCCTAATCCCCCTGGC |
|  | PN18-13-F | TTAGAATAGCGGCGATGG | PN18-13-R | CTCGGCAGCAATGAAGAC |
|  | PN15-96-F | CTCATAAGGGAATCGTGG | PN15-96-R | TTGACATCTCGCACTACC |
|  | PN15-96nei-F | AGCTTCAGCAGGACACTC | PN15-96nei-R | CCCAAGGGAAATCAGCAG |
|  | PN-Z1 | CTCCTACTTCAGGTCCAT |  |  |
|  |  |  |  |  |
| *Samaris cristatus* | GD2 -6468-Z | TCTACAATCCACCGCAATCG | GD2 -6468-F | CCGAATCCTCCAATCAAG |
|  | GD2 -6468-Z-2 | TACTGGCATCTGGTTCCT | GD2 -6468-F-2 | TGCCGATGTCTTTGTGGTTG |
|  | GD2-Z-2323 | CATAAGACGAGAAGACCCTG | F-COI-5end | TTTCCGAATCCTCCAATC |
|  | GD2-Cytb-f | AGTTTCATCAAGCGGAGA | GD2-Cytb-z | CGCTAACGATGCTCTAAT |
|  | Z10818 | TTYGAAGCAGCCGCMTGATACTGACAYTT | GD2-ND5-F | TATTGCGGAGGGTAGTCA |
|  | L-REPEATNC | AACGGAGGGAAGATAATG | GD2-F755 | TACGCTACACCTTGACCTGA |
|  | GD2-Z-ND4 | CAACCCGAGAACACCTAA |  |  |
|  |  |  |  |  |
| *Achirus lineatus* | AL-Pro | TTCCACCTCTAACTCCCAAAGCTAG | AL-Phe | CCCATCTTAACATCTTCAGTG |
|  | AL-Phe | CTGAAGATGTTAAGATGGG | AL-16SAR | ATGTTTTTGATAAACAGGCG |
|  | AL-16SAR | CGCCTGTTTATCAAAAACAT | AL-16SBR | CCGGTCTGAACTCAGATCACGT |
|  | AL-123 | ATCAACGAACCAAGTTATCCTA | AL-123 | TCGGGGAGTCAAGAGAGT |
|  | AL-ND2 | ACTTCTAGCTTGAATAGGCCT | AL-ND2 | AGTTAAGCGGTGGATTGTAG |
|  | AL-COI | TCTAGATAGGAAGGCCTTGAT | AL-COI | AGAAAGTGACAGAGCGGTTAT |
|  | AL-CAA | CACCCTTTGAAACACTATCTC | AL-CAA | AGTGGAATCAGATAGCAAGG |
|  | AL-ND34 | CCAAGTGAGATAATGAGCC | AL-ND34 | CCTAAGACCAACGTATGAGC |
|  | AL-ND56 | GTTGAACTGCCTGTAGGTGTA | AL-ND56 | TGTCCTGGTTTTAGTCCTGG |
|  | AL-CytB | GTATATTAAGGCCACTCCTC | AL-CytB | CTGATGAGTGTTGTGTTCGG |
|  |  |  |  |  |
| *Trinectes maculatus* | TM-Z11730 | AAAGCCCGAACTTGTCTG | TM-F12120 | CTGGTGAAGAAGGCTAAT |
|  | TM-Z7350 | TGCCATAGTCTCAGCCAAAC | TM-F9860 | TTGCGACGAGAAAGAAGC |
|  | TM-Z-650 | ACCTCACCCTTCCTTGTT | TM-F-2050 | GTAGCAAGAGGCGATGTT |
|  | TM-Z-15420 | TTTCTGACTCCTGGTTGC | TM-F-16060 | GGTATGACGGGATGTTGA |
|  | TM11-Z-15 | TTTTCCACTACTAACCCC | TM11-F-650 | TCTGGGCTGTGCCGATTT |
|  | TM-Z-650 | AAGTAAGCCAGAAATAGAGCG | TM-COI | AGAAAGTGACAGAGCGGTTAT |
|  | TM-CAA | TCCTCCCCAAAACAAATG | TM-CAA | GGCTTACTTCTAAATCCTCCT |
|  | TM-ND34 | CCAAGTGAGATAATGAGCC | TM14-F-2753 | CCTAAGACCAACGTATGAGC |
|  | TM-ND56 | GTTGAACTGCCTGTAGGTGTA | TM-ND56 | TGTCCTGGTTTTAGTCCTGG |
|  | TM-L-CytB | GTATATTAAGGCCACTCCTC | TM-CytB | CTGATGAGTGTTGTGTTCGG |
|  |  |  |  |  |
| *Cynoglossus nanhaiensis* | Z15 | ATTAAAGCATAACHCTGAAGATGTTAAGAT | F2671 | AGATAGAAACTGACCTGGAT |
|  | 16SAR | CGCCTGTTTATCAAAAACAT | F5196 | CTGGAAGCACGAAGAGTT |
|  | PN-COI-R | ACACCGCCTTCTTTGACC | F11089 | ATCCAGGTCAGTTTCTATC |
|  | FishR1 | TCAACCAACCACAAAGACATTGGCAC | FishF1 | TAGACTTCTGGGTGGCCAAACAATCA |
|  | PN15-96-R | TTGACATCTCGCACTACC | PN-COI-F | AGGTTGGCTAAGTTCGGC |
|  | Z10818 | TTYGAAGCAGCCGCMTGATACTGACAYTT | F13413 | TAGCTGCTACTCGGATTTGCACCAAGAGT |
|  | L14734 | AACCACCGTTGTTATTCAACT | F17147 | TAGTTTARTGCGAGAATCCTAGCTTTGGG |
|  | PN18-13-R | CTCGGCAGCAATGAAGAC | PN34-47-F | AGCACCTAAGCAGAGTCC |
|  | CM-Z-Thr-1 | CCTTGTAAGTCGGATGCC | F6746 | GCGGTGGATTGTAGACCCATARACAGAGGT |
|  | Z-COM-Tyr-19 | ACCTCYRTHBDYRGRDYTACAAYCC | F-COM-Phe-19 | GTTATGCTTTRNWTAAGCTAC |
