# Supplementary material for: Flatfish monophyly refereed by the relationship of Psettodes in Carangimorphariae
Source: BMC Genomics. 2018 May 25;19:400. doi: 10.1186/s12864-018-4788-5 (PMC5970519; doi:10.1186/s12864-018-4788-5)
Supplement: Supplementary file 2 — Figure S1. a Relationships of Carangimorphariae taxa inferred from the Bayesian analysis v3.2 of the 50 taxa of dataset 1N2N. Numbers above or below the branches indicate Bayesian posterior probabilities (shown as percentages). b Relationships of Carangimorphariae taxa inferred from the Bayesian analysis version 3.2 of 50 taxa of dataset 1N2N3RY. Numbers above or below internal branches indicate Bayesian posterior probabilities (shown as percentages). c Relationships of Carangimorphariae taxa inferred from the Bayesian analysis version 3.2 of the 50 taxa of dataset 1N2NRT. Numbers above or below internal branches indicate Bayesian posterior probabilities (shown as percentages). d Relationships of Carangimorphariae taxa inferred from the Bayesian analysis version 3.2 of 50 taxa of dataset 1N2N3RYRT. Numbers above or below internal branches indicate Bayesian posterior probabilities (shown as percentages). e A maximum likelihood (ML) tree generated in RAxML version 8.0.0 under a GTR + Γ model of nucleotide evolution. The 50 taxa mitogenomes were partitioned by codon position (1N2N). f A maximum likelihood (ML) tree generated in RAxML version 8.0.0 under a GTR + Γ model of nucleotide evolution. The 50 taxa mitogenomes were partitioned by codon position with the third codons recoded (1N2N3RY). g A maximum likelihood (ML) tree generated in RAxML version 8.0.0 under a GTR + Γ model of nucleotide evolution. The 50 taxa mitogenomes were partitioned by codon position, rRNA, and tRNA (1N2NRT). (DOCX 2386 kb) [file 12864_2018_4788_MOESM2_ESM.docx]

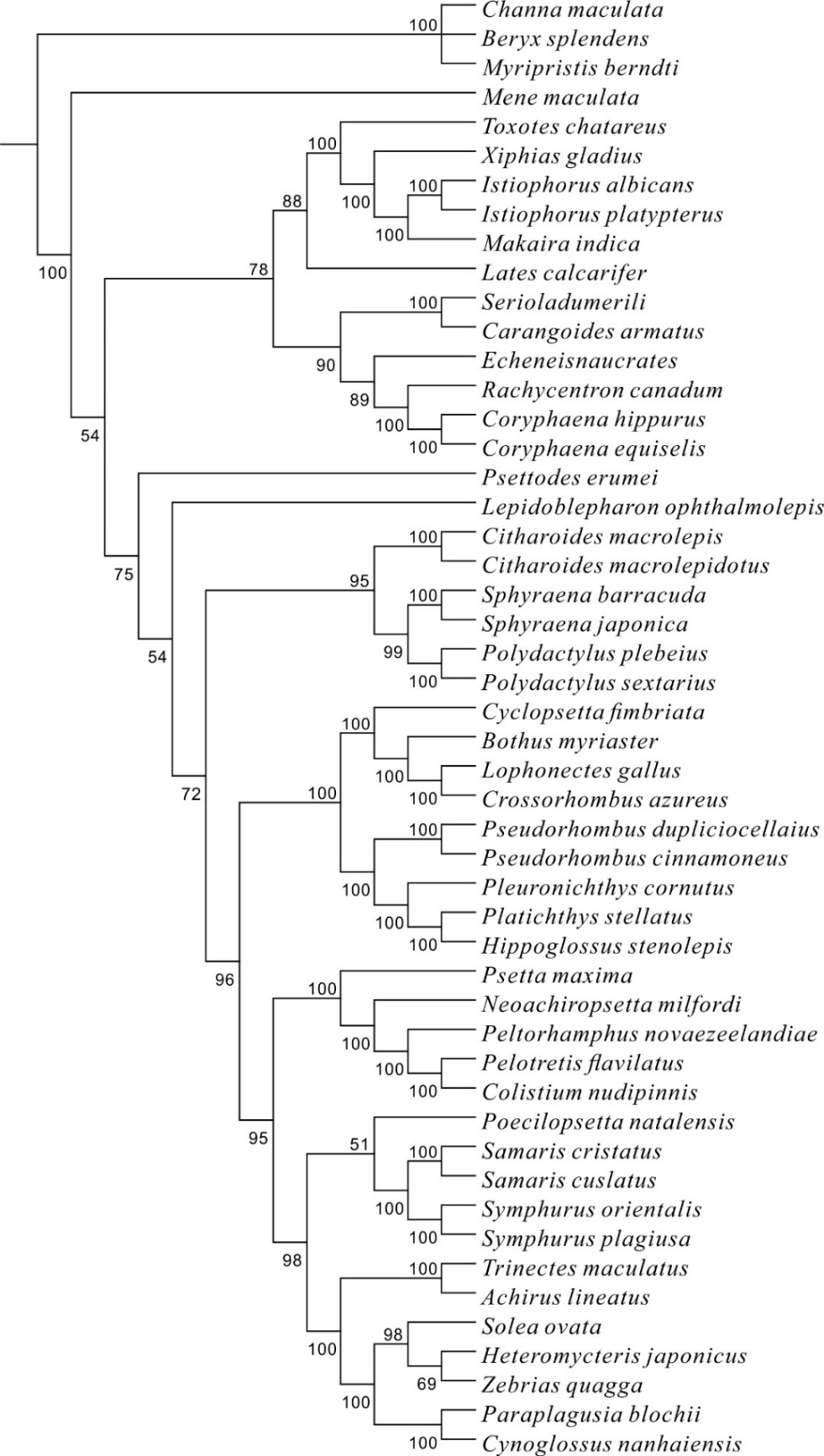


Figure.S1a

Relationships of Carangimorphariae yielded in the Bayesian analysis version 3.2 from the 50 taxa of dataset 1_N_2_N_. Numbers above or under the branches indicated Bayesian posterior probabilities (shown as percentages).


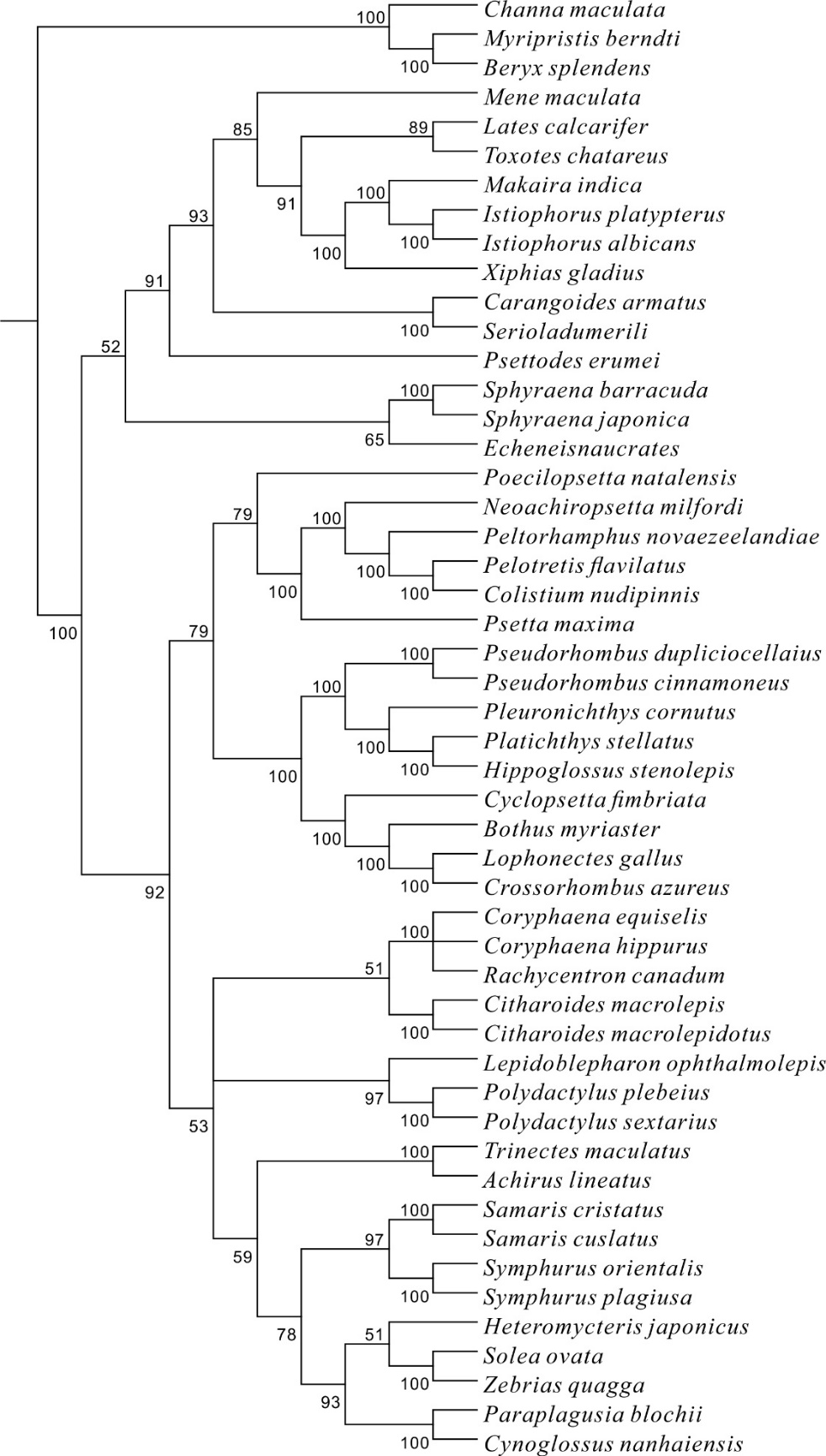


Figure.S1b

Relationships of Carangimorphariae yielded in the Bayesian analysis version 3.2 from 50 taxa of dataset 1_N_2_N_3_RY_. Numbers above or under internal branches indicated Bayesian posterior probabilities (shown as percentages).


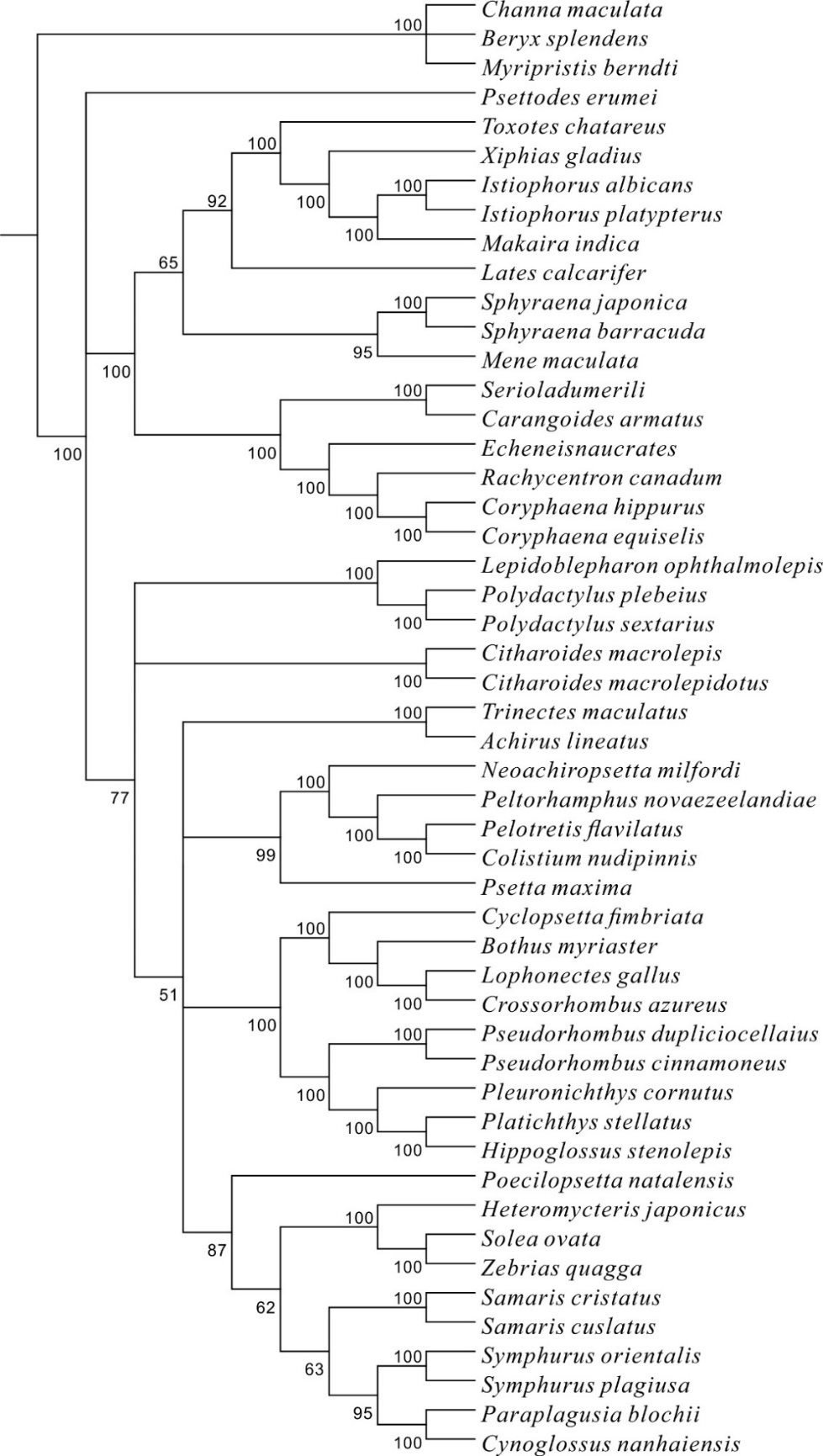


Figure.S1c

Relationships of Carangimorphariae yielded in the Bayesian analysis version 3.2 from the 50 taxa of dataset 1_N_2_N_RT. Numbers above or under internal branches indicated Bayesian posterior probabilities (shown as percentages).


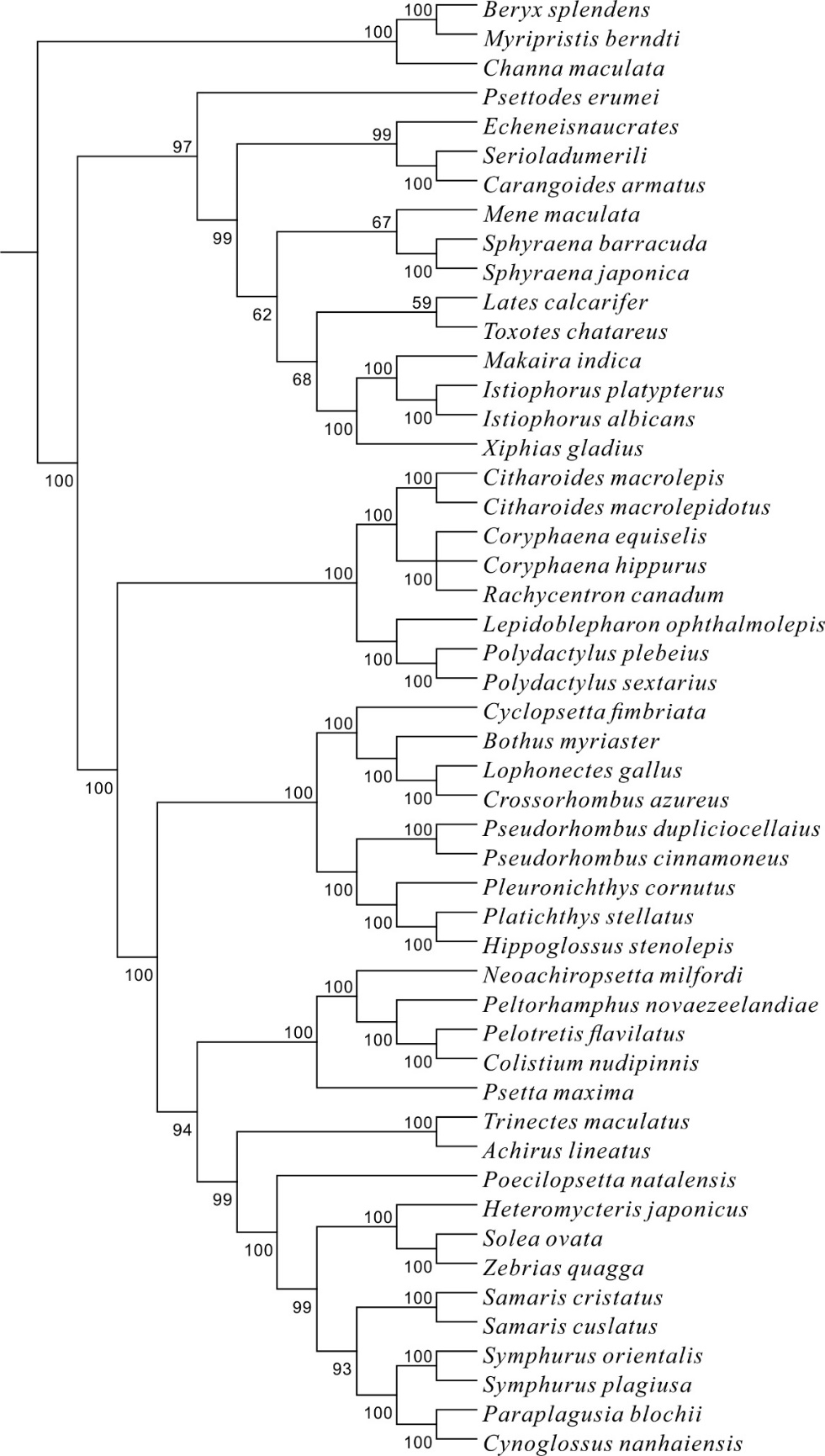


Figure.S1d

Relationships of Carangimorphariae yielded in the Bayesian analysis version 3.2 from 50 taxa of dataset 1_N_2_N_3_RY_RT. Numbers above or under internal branches indicated Bayesian posterior probabilities (shown as percentages).


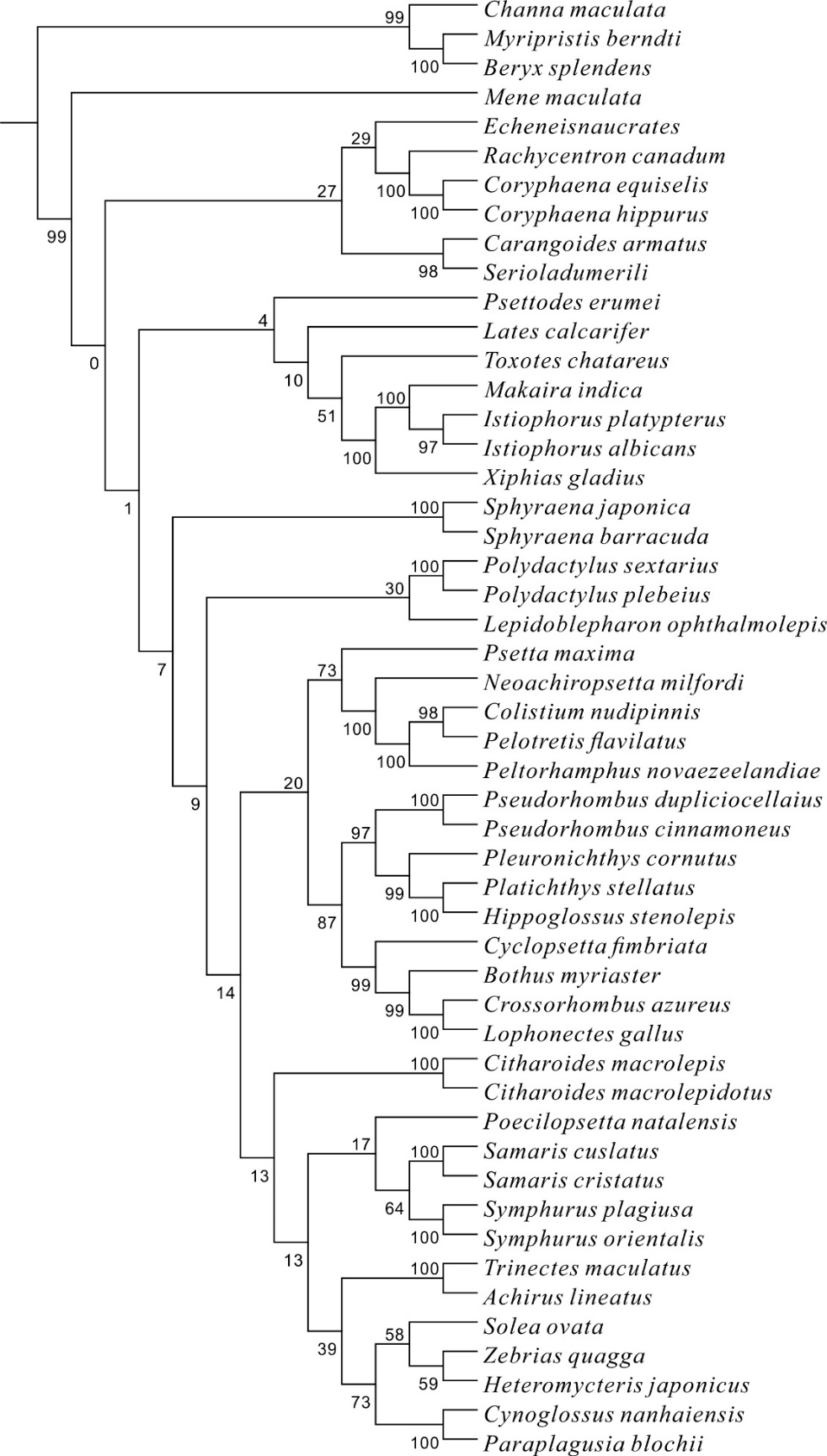


Figure.S1e

A maximum likelihood (ML) tree generated in RAxML version 8.0.0 under a GTR+Γ model of nucleotide evolution. The 50 taxa Mitogenomes were partitioned by codon position (1_N_2_N_).


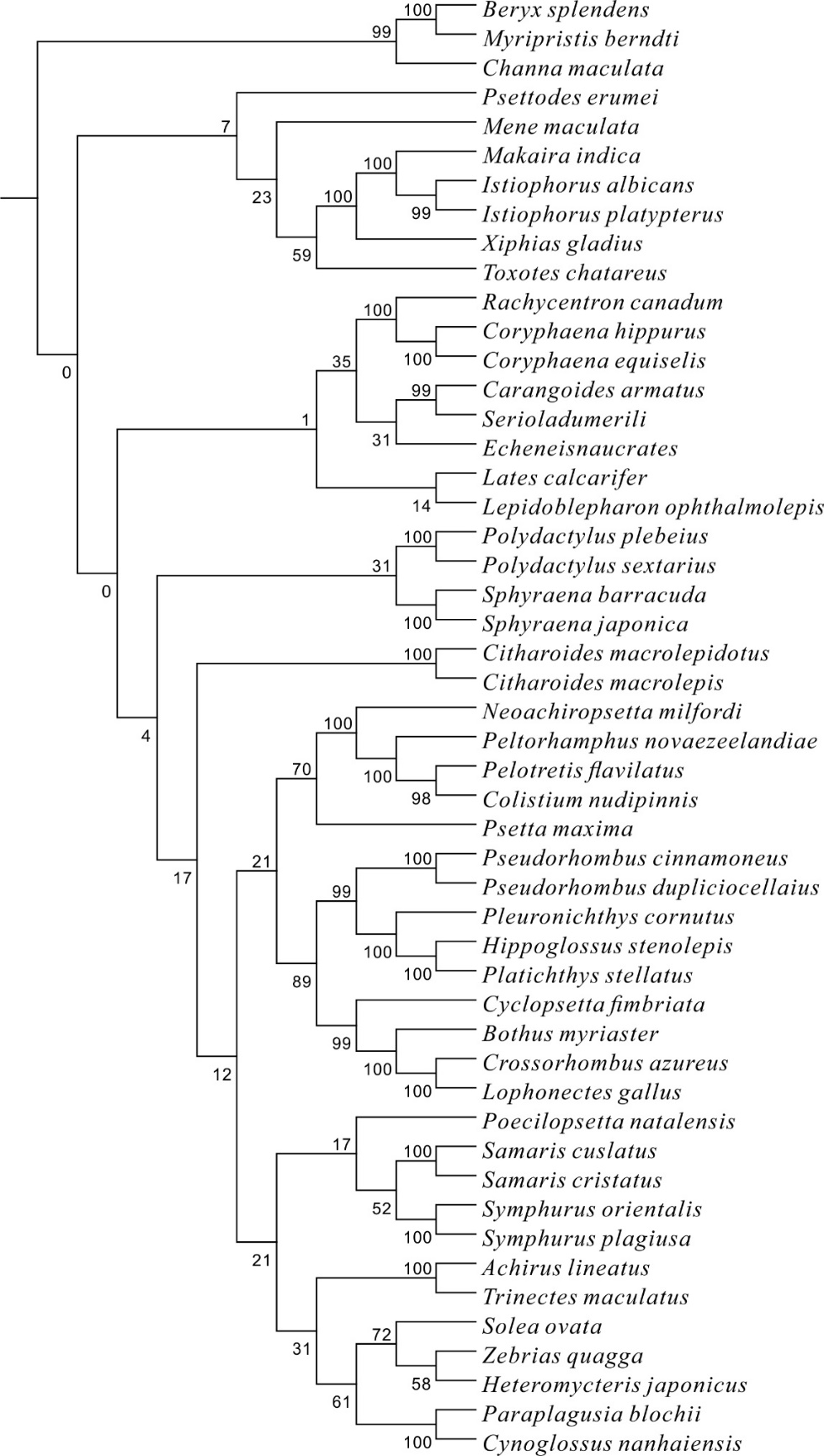


Figure.S1f

A maximum likelihood (ML) tree generated in RAxML version 8.0.0 under a GTR+Γ model of nucleotide evolution. The 50 taxa Mitogenomes were partitioned by codon position with third codons recoded (1_N_2_N_3_RY_).


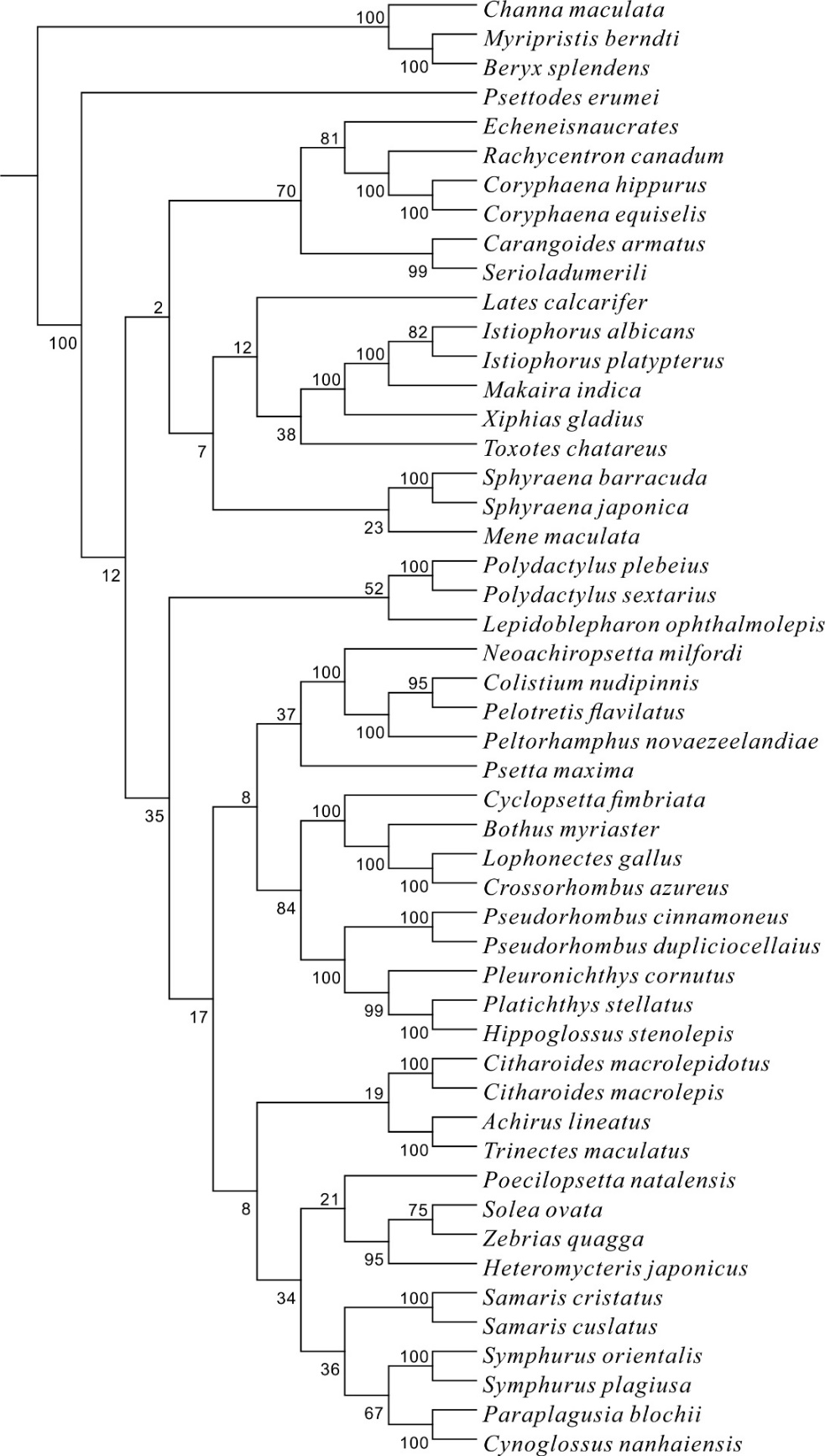


Figure.S1g

A maximum likelihood (ML) tree generated in RAxML version 8.0.0 under a GTR+Γ model of nucleotide evolution. The 50 taxa Mitogenomes were partitioned by codon position, rRNA, and tRNA (1_N_2_N_RT).
